# Prognostic value of preoperative lymphocyte-to-monocyte ratio in gallbladder carcinoma patients and the establishment of a prognostic nomogram

**DOI:** 10.1097/MD.0000000000021021

**Published:** 2020-07-31

**Authors:** Yan Deng, Ming-Fang Xu, Feng Zhang, Xiao Yu, Xue-Wen Zhang, Zhen-Gang Sun, Shuai Wang

**Affiliations:** a Department of Hepatobiliary Surgery; b Department of Otolaryngology; c Department of Ophthalmology, Jing Zhou Central Hospital, The Second Clinical Medical College, Yangtze University, Jing Zhou, Hubei, China.

**Keywords:** gallbladder carcinoma, lymphocyte-to-monocyte ratio, nomogram, prognosis

## Abstract

The purpose of this study was to investigate the potential prognostic value of preoperative lymphocyte-to-monocyte ratio (LMR) and establishment of a prognostic nomogram in post surgical patients with gallbladder carcinoma (GBC).

Receiver operating characteristic curve analysis was performed to determine the optimal cut-off value of LMR. The correlation between preoperative LMR and overall survival (OS) was analyzed using univariate and multivariate Cox regression analyses. A relevant prognostic nomogram was established.

Three hundred fifteen GBC patients were retrospectively enrolled. Based on receiver operating characteristic curve analysis, the optimal cutoff value of LMR was 2.685. Patients were categorized into high-LMR group (n = 143) or low-LMR group (n = 172). Low-LMR value was significantly associated with elderly age, advanced tumor, and the performance of a palliative cholecystectomy. The results of the univariate and multivariate analyses eliminated the degree of tumor differentiation, tumor-node-metastasis stages, surgery types, and LMR as independent predictors of OS. Based on those independent predictors, a predictive nomogram for OS was generated with an accuracy of 0.848.

Based on our findings, the predictive nomogram should be included in the routine assessment of GBC patients.

## 1 Introduction

In the US, gallbladder carcinoma (GBC) is the fifth most common gastrointestinal malignancy of the 21st century.^[1,2]^ The incidence of GBC has been calculated to be 2.5 per 100,000 persons based on the data obtained by the Epidemiology and End Results program.^[1,3]^ However, GBC has an abysmal prognosis. The poor prognosis of patients with GBC is largely due to the aggressive biological behavior of this disease, its vague and nonspecific symptom, and the aberrant anatomical feature of gallbladder in which the wall adjacent to the liver lacks a serosal layer.^[4,5]^ There is no doubt that a complete resection is still the most effective treatment measure, if allowed by the condition of patient.^[6]^ Palliation is generally the fundamental treatment for patient with symptomatically advanced GBC. However, the prognosis of certain patients who have undergone a radical surgical resection remains appalling.^[7]^ Classifying patients can facilitate predicting their overall survival (OS) period and thus aid in selecting appropriate therapies that might improve their clinical outcomes.^[8,9]^ Therefore, the prognostic determinants of the risk of mortality must be more finely delineated to allow better stratification of the patients who are likely to reap large rewards from surgical treatment.

The relationship between inflammation and the oncogenesis of various types of malignant cancers has been characterized.^[10]^ Exploring the contribution of inflammation to the development of those cancers is an area of ongoing investigation, particularly in regions where cancers are more prevalent.^[11]^ Mounting evidence suggested that presence of inflammation, as reflected in routine blood counts, has a decidedly pro-tumor effect and suppresses the host immune system, which leads to a poor prognosis for patients with a host of malignant neoplasms.^[12]^ Additionally, the number of observed blood cells associated with inflammation and immunity, including neutrophils, lymphocytes, and monocytes, might be affected by the tumor itself.

Lymphocyte-to-monocyte ratio (LMR) is calculated by dividing the lymphocyte count by the monocyte count. High LMR indicates a higher lymphocyte count and a lower monocyte count. Lymphocyte act as a tumor suppressor by inhibiting tumor cell proliferation and migration. In contrast, monocyte suppresses the host's antitumor immune response and promote inflammation. An association between the LMR value and the prognosis for various types of malignant neoplasms has been found.^[8,13–21]^ However, to the best of our knowledge, the effect of LMR on GBC patients has not been investigated. This study was therefore designed to evaluate the prognostic significance of pretreatment LMR in GBC patients who had undergone a radical or a palliative cholecystectomy, and attempt to establish a prognostic nomogram with improved predictive capacity in these patients.

## 2 Materials and methods

### 2.1 Patients

A total of 390 GBC patients who had presented to 1 team from the Jing Zhou Central Hospital, the Second Clinical Medical College, Yangtze University between 2001 and 2017 were enrolled in this retrospective study. As described by Kanthan et al,^[22]^ these patients received various surgical treatments. Each of the patients had undergone a radical or palliative cholecystectomy, and their tumor specimens had been pathologically and histologically confirmed to be GBC. The patients excluded from the study met one of the following criteria:

(1) having multiple cancers;
(2) incomplete peripheral blood lymphocyte or monocyte count data; and
(3) having a systemic infection, autoimmune disease, or inflammation.

We also excluded 31 patients with incomplete follow-up data. At last, 315 patients remained and were analyzed in this study. Informed consent was obtained from all the enrolled patients, and the study was approved by the Ethical Committee of Jing Zhou Central Hospital, the Second Clinical Medical College, Yangtze University and the methods were carried out in accordance with the relevant guidelines and regulations. In addition, the patient record/data was anonymized and was de-identified before analysis. Besides, informed consent was obtained from all patients or their family members.

### 2.2 Data collection

Medical information for all the patients was collected from clinical records, including the demographic data (age and sex), the surgical procedure performed, the presence of a concomitant disease (hypertension, diabetes mellitus or cystic liver), the tumor-node-metastasis (TNM) stage, and the pathological reports. In addition, the data obtained from blood tests, including the blood type, hemoglobin level, and peripheral blood lymphocyte and monocyte counts, were collected. Preoperative anemia was defined as having a baseline hemoglobin level of <120 g/L for males or <110 g/L for females. Histopathology and clinical staging were performed through postoperative histopathological examination and clinical assessment according to the guidelines of the American Joint Committee on Cancer (8th edition), respectively. Blood samples were extracted from peripheral blood test before surgery. If more than 1 set of blood samples were obtained from the same patient, the earliest set of results was utilized in this study. The LMR was determined by dividing the lymphocyte count by the monocyte count.

### 2.3 Follow-up

All the patients were routinely followed-up postoperatively every 3 months for the first year, every 4 months for the second year, and every 6 months thereafter, until November 2017. The endpoint of this study was the OS during the interval between surgery and death or during the interval between surgery and the last follow-up. The post-treatment surveillance program consisted of a physical examination, a cytological assessment, and ultrasonic or abdominal computed tomography. During the follow-up, the postmortem interval and the details of the imaging results were recorded. To maximally reduce the extent of bias, Two specific clinicians in our hospital performed the follow-up and the review. Finally, all relevant data including follow-up information was restored in archives room.

### 2.4 Statistical analysis

Operating characteristic curve (ROC) analysis was used to determine the optimal cut-off value for predicting the 5-year OS with the best level of sensitivity and specificity. Based on this cut-off value, the patients were divided into 2 groups, namely low-LMR group and high-LMR group. In the case of continuous variables, the data were expressed as the mean value ± standard deviation or as the median (min-max) values, depending on whether they had a normal distribution (Kolmogorov–Smirnov test, *P* < .050). The categorical variables were presented as frequency distributions. Accordingly, the significance of the differences was determined using Student *t* test or the Wilcoxon test for continuous variables and the Chi-squared test for categorical variables. OS values of high- and LMR groups were calculated using the Kaplan–Meier (KM) method and were compared using the log-rank test. Variables shown to have significant prognostic value using univariate analysis were further analyzed using the multivariate Cox proportional hazards model. Hazard ratios (HRs) and the corresponding 95% confidence intervals (CIs) were determined using Cox regression analysis. A nomogram for possible prognostic characteristics associated with OS was performed by R software version 3.3.1 using the package of rms. Calibration Plots were performed to examine the performance characteristics of the nomogram. The Harrell concordance index was used to assess its predictive accuracy. All the statistical analyses were performed using PASW Statistics 22.0 software (SPSS Inc., Chicago, IL) and R version 3.3.1 software (Vienna, Austria). Two-sided *P*-values of less than .05 were considered to indicate significant differences.

## 3 Results

### 3.1 Patient characteristics

The baseline clinical and pathological characteristics of 315 patients with a diagnosis of GBC are shown in Table 1. The median age of patients was 64 years (range 30–87), 67.94% females, and 50.48% had a previous history of gallstones. Based on the TNM staging system, 17 patients were diagnosed with a stage I tumor, 42 with a stage II tumor, 90 with a stage III tumor, and 163 with a stage IV tumor. Regarding the pathological differentiation levels of the tumors, 50.16%, 39.68%, and 10.16% of tumors were poorly differentiated, moderately differentiated, and well differentiated, respectively. Most of patients had undergone a radical cholecystectomy (53.65%), whereas the rest had undergone a palliative cholecystectomy (46.35%). A minority of patients had concomitant diabetes mellitus, hypertension or a cystic liver.

**Table 1:**
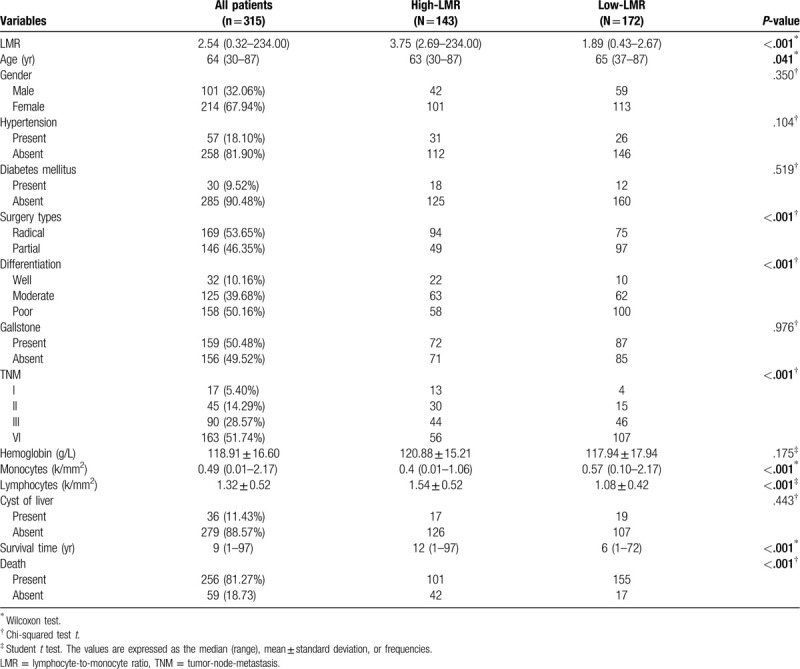
Baseline characteristics of the patients with GBC according to pretreatment LMR.

### 3.2 ROC curve analysis

**Figure 1:**
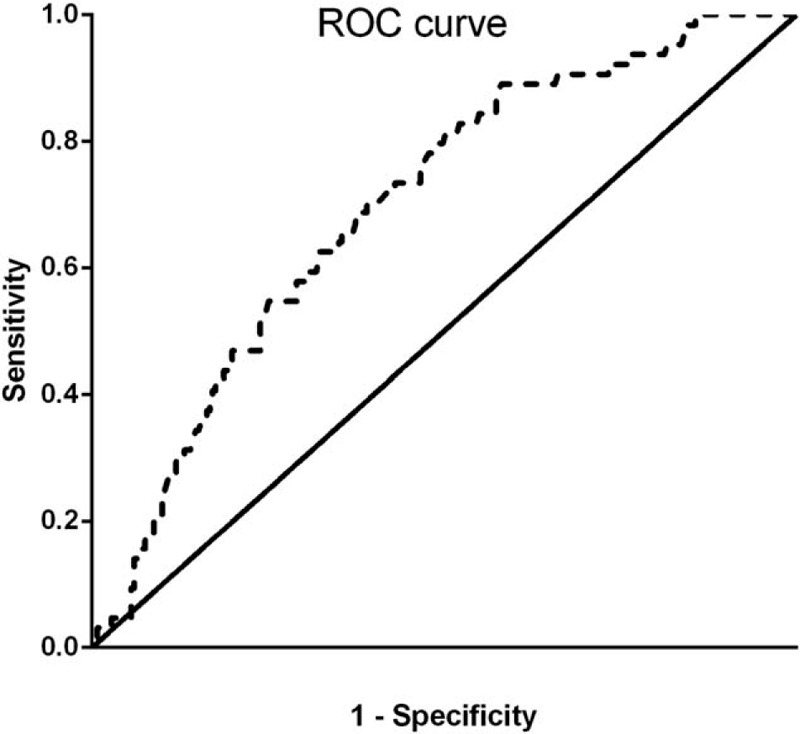
A receiver operating characteristic (ROC) curve for the prediction of the overall survival (OS) period was plotted to determine the optimal cut-off points for the LMR. LMR = lymphocyte-to-monocyte ratio.

ROC curve analysis was performed to determine the optimal cut-off value for LMR based on 5-year OS prediction. As shown in Figure 1, the optimal cut-off value for LMR was determined to be 2.685. The area under the curve was 0.698 (95% CI: 0.631–0.766, *P* < .001), with a specificity of 61.01% and a sensitivity of 70.30%. Based on the cut-off value, patients were categorized into 2 groups, namely high-LMR group (≥2.685) group and low-LMR (<2.685) group. There were 143 (45.40%) patients with a high preoperative LMR and 172 (54.60%) patients with a low preoperative LMR.

### 3.3 The correlation of the LMR with the clinicopathological features

**Figure 2:**
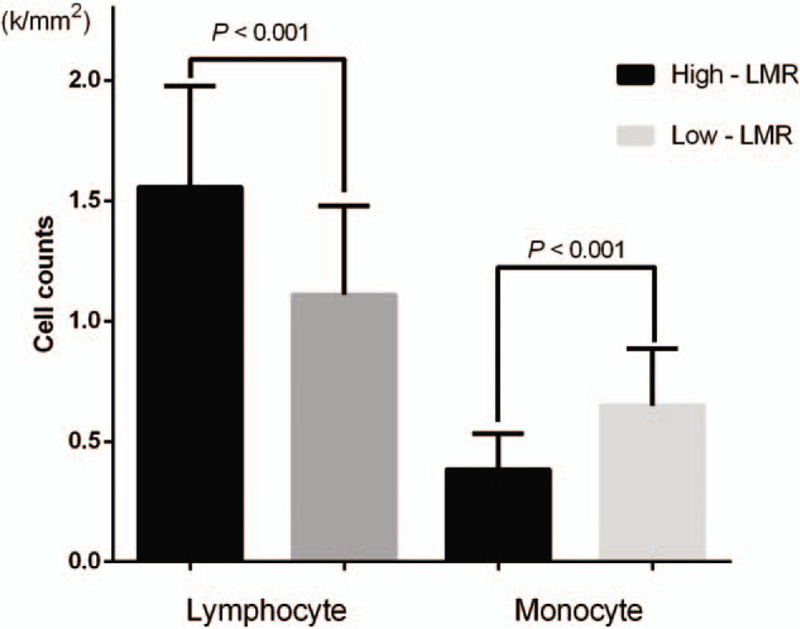
Histogram comparing the lymphocyte and monocyte counts of GBC patients with a high LMR and a low LMR (*P* < .001). GBC = gallbladder carcinoma, LMR = lymphocyte-to-monocyte ratio.

To probe the correlations between preoperative LMR of GBC patients and their clinicopathological features, the 2 groups were compared (Table 1). Compared with patients in the high-LMR group, patients in the low-LMR group had significantly more advanced tumors, including tumors with a poor level of differentiation (*P* < .001) and an advanced TNM stages (*P* < .001). Moreover, significantly more elderly patients were in the low-LMR group. There was a significant difference between the types of surgery performed on the study participants in the low- and high-LMR groups. The patients in the low-LMR group had a lower rate of treatment with radical cholecystectomy compared with those in the high-LMR group (*P* < .001). The distributions of the lymphocyte and monocyte counts in the 2 groups are shown in Figure 2. The participants with a high LMR had a lower monocyte count and a higher lymphocyte count (*P* < .001). No significant differences in the other clinicopathological variables of the 2 groups were found.

### 3.4 Univariate analysis of the predictors of mortality

**Figure 3:**
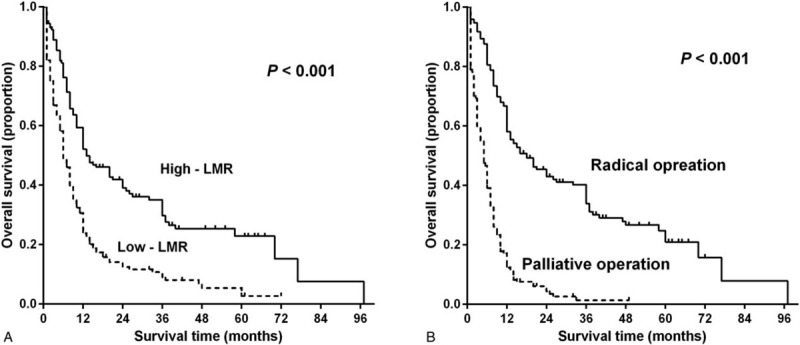
Kaplan–Meier survival curves predicting the overall survival of (A) groups categorized according to the preoperative lymphocyte to monocyte ratio (*P* < .001) and (B) groups stratified according to the different surgical procedures performed (*P* < .001). The *P*-values were calculated using the log-rank test.

At the end of the study, 256 patients (81.27%) had died. The median post-operative OS period was 9 months (range: 1–97 months). The postoperative patients had 1-, 3-, and 5-year probabilities of mortality of 37.1%, 17.8%, and 11.4%, respectively. One-hundred one of the 143 high-LMR patients died (70.63%) and 155 of 172 low-LMR patients died (90.12%) during the follow-up period. The patients in the high-LMR group had lower 1- (52.4% vs 23.8%), 3- (30.1% vs 18.5%), and 5-year (23.1% vs 2.7%) mortality rates compared with those of the low-LMR group (*P* < .001 for each dataset). As shown in KM curve in Figure 3A, the 5-year OS rate of low-LMR group was significantly lower than that of high-LMR group (*P* < .001).

Subsequently, using univariate Cox regression analysis, a higher risk of mortality was found to be associated with patients according to the type of surgery performed [HR: 3.796; 95% CI: 2.903–4.964; *P* < .001], the degree of tumor differentiation [HR: 1.795; 95% CI: 1.478–2.180; *P* < .001], the TNM stage [HR: 2.353; 95% CI: 1.978–2.798; *P* < .001], the co-occurrence of a cystic liver [HR:1.784; 95% CI: 1.130–2.818; *P* = .013], the co-occurrence of anemia [HR:1.305; 95% CI: 1.011–1.686; *P* = .041] and the LMR [HR: 0.465; 95% CI: 0.360–0.601; *P* < .001] (Table 2).

**Table 2:**
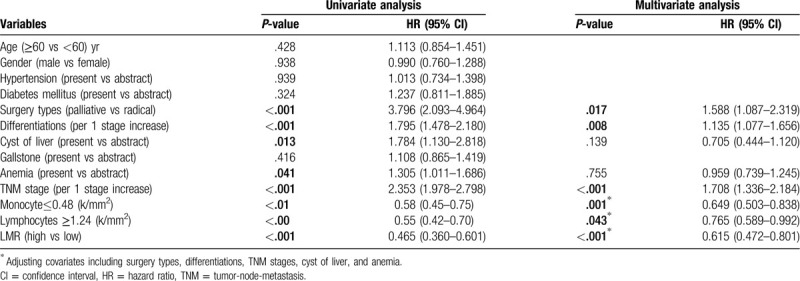
Univariate and multivariate analysis of variables associated with GBC patients OS.

### 3.5 Multivariate analysis of the predictors of mortality

To avoid the occurrence of collinearity of TNM stage with T stage, N stage, and M stage, the TNM stage was not enrolled into the Cox regression modeling because it was calculated based on T stage, N stage, and M stage. Multivariate analysis using the Cox proportional hazard model screened out the degree of tumor differentiation [HR: 1.135; 95% CI: 1.077–1.656; *P* = .008], TNM stage [HR: 1.708; 95% CI: 1.336–2.184; *P* < .001], surgery types [HR: 1.588; 95% CI: 1.087–2.319; *P* = .017], and the LMR value [HR: 0.615; 95% CI: 0.472–0.801; *P* < .001] as independent predictors of the OS period (Table 2).

### 3.6 Additional analyses

**Figure 4:**
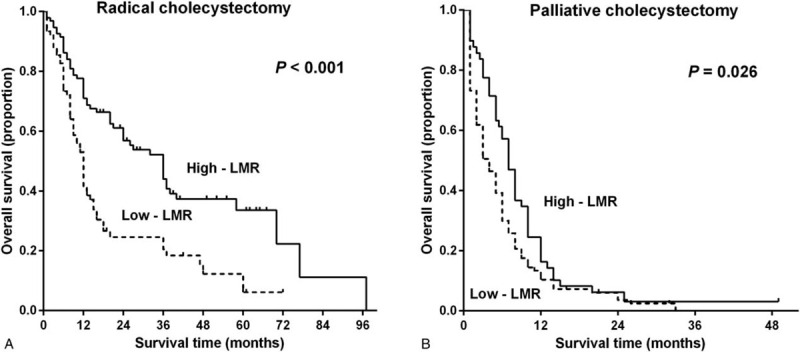
Kaplan–Meier curves of the overall survival periods of patients in the low and high preoperative lymphocyte to monocyte ratio subgroups of the different surgical-procedure groups. (A) The radical cholecystectomy group (*P* < .001) and (B) the palliative cholecystectomy group (*P* = .026). The *P*-values were calculated using the log-rank test.

Consistent with the results of prior investigations,^[23]^ in this study, the surgical procedure type was revealed to be associated with postoperative mortality risk (Fig. 3B). To determine whether the association of the LMR with the prognosis that was described above was merely secondary to the surgical procedure performed, patients were classified according to the specific type of surgical intervention to which they were subjected to form a radical cholecystectomy group (n = 169) and a palliative cholecystectomy group (n = 146). KM curves showed that low-LMR subgroups in both surgical-procedure groups had significantly higher mortality rates compared with those of the high-LMR subgroup (Fig. 4A and B).

**Figure 5:**
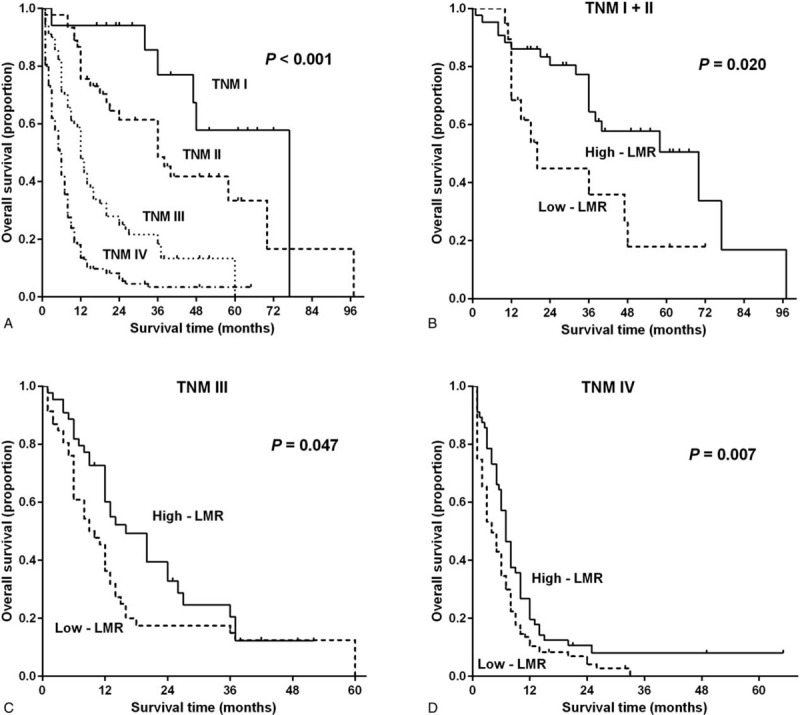
(A) Kaplan–Meier survival curves for patients according to the TNM stage of their tumors (*P* < .001). Kaplan–Meier survival curves for patients in the low and high preoperative lymphocyte to monocyte ratio subgroups of the different TNM-stage groups, including the (B) TNM I + II group (*P* = .020), (C) the TNM III group (*P* = .047), and (D) the TNM IV group (*P* = .007). The *P*-values were calculated using the log-rank test. TNM = tumor-node-metastasis.

The TNM stage of various types of malignancies was found to be a potent predictor of clinical outcomes,^[24,25]^ which was corroborated by the results of this study (Fig. 5A). To clarify whether the TNM stage had a negative effect on the OS of the GBC patients, all the enrolled patients were stratified based on the different TNM stages of their tumors. Analysis of these data showed that the OS periods of the high-LMR subgroups of the stage I + II (*P* = .020), stage III- (*P* = .047), and stage IV TMN groups were longer than those of the low-LMR subgroups (*P* = .007) (Fig. 5B–D).

### 3.7 Prognostic nomogram for OS

**Figure 6:**
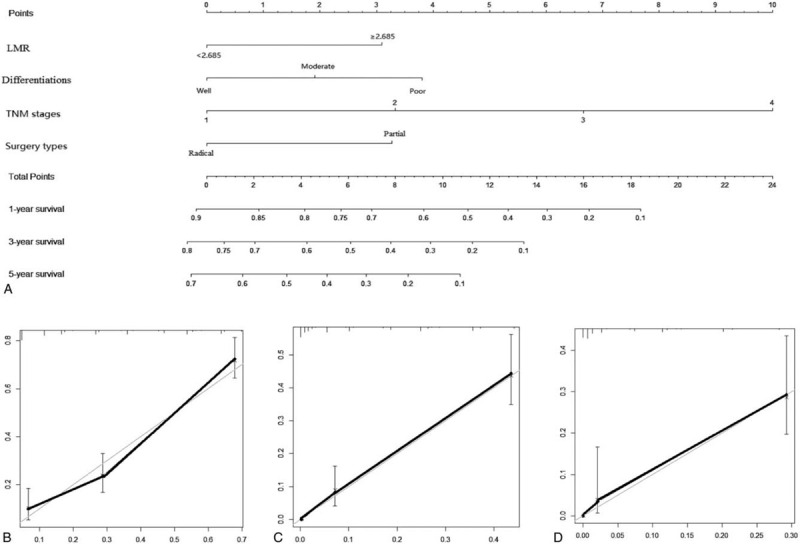
(A) Nomogram for predicting 1-, 3-, and 5-yr OS of GBC patients after surgery. A line was drawn upward to confirm the number of points received for each independent indicator and the points for each parameter is located on the top of point axis. Moreover, the sum of total points was located on the total point axis, a body line was drawn upward to the survival axes to determine the rate of 1-, 3-, and 5-yr OS, respectively. Calibration plots of the nomogram for predicting 1-(B), 3-(C), and 5-(D) yr survival. The fine line represents the ideal line of a perfect match between the predication by the nomogram and actual observation. GBC = gallbladder carcinoma, OS = overall survival.

To further predict OS of GBC patients after surgery, a predictive nomogram was depicted by COX regression analysis using all the significant independent factors for OS consisting of T NM stages, surgery types, the degree of tumor differentiation, and LMR (Fig. 6A). A nomogram was used by totaling each point identified on the top scale for each independent factor and a higher total point indicates a reduced OS. It can predict the probability of death of GBC within 1, 3, or 5 years after surgery. The calibration plots for the probability of 1-, 3-, and 5-year survival showed an optimal agreement between the prediction by nomogram and actual observation (Fig. 6C and D). The concordance index of the multivariate prognostic model was 0.848 and reduced to 0.773 when the LMR was removed.

## 4 Discussion

Although ongoing improvements in diagnostic and surgical techniques have resulted in a major decline in the mortality rate of patients with GBC, the prognosis for GBC remains dismal due to the aggressive biological behavior of this malignancy and the lack of sensitive screening tests allowing its early detection.^[26,27]^ Tumor-related factors, such as the TNM stage and the degree of histological differentiation, have been shown to be predictors of the clinical outcome.^[28]^ However, those particular tumor-related factors reflect only the degree of disease progression and only partially explain the prognostic heterogeneity.^[29]^ With advances in understanding tumor biology, there is increasing evidence that an infiltrating inflammatory microenvironment and a compromised immunological status are significant determinants of the post-treatment outcome. Hence, identifying an objective and efficacious predictor of the clinical outcome regarding the host immunological status and inflammatory response is critically important.

Clearly, malignant patients’ clinical outcome is determined not solely by tumor characteristics that only reflect the degree of cancer progression, but also by host-related factors such as host response to systemic inflammation. Moreover, LMR could indicate the body's immunity, inflammation, and nutrition status at the same time. Hence, we expected that a combination of host-related factors with conventional tumor characteristics could accurately predict individualized OS in GBC patients after surgery. LMR has been confirmed to be a significant prognostic index for various types of malignant neoplasms However, few studies have investigated the effect of the preoperative LMR on the clinical outcome of patients with GBC. In the present study, we retrospectively analyzed a large cohort of 315 patients with a diagnosis of GBC and found a significant association between LMR and OS using univariate analysis. Via multivariate analysis, LMR was found to be an independent advantageous prognostic factor, even after correcting for the clinicopathological features. In the subgroup analyses, the association between LMR and OS was found to apply to both patients who had undergone a radical cholecystectomy and patients who had undergone a palliative cholecystectomy, as well as to patients with tumors of different TNM stages. Therefore, our study demonstrated that the prognostic value of preoperative LMR was independent of the traditional prognostic predictors. Furthermore, a predictive nomogram was depicted by COX regression analysis using all the significant independent factors for OS and Calibration plots of the nomogram performed well in the prediction of OS with an accuracy of 0.848.

Although the LMR has been used in other investigations to evaluate the clinical outcomes of patients with various types of malignancies, there is no uniform optimal cut-off value for LMR. To determine the optimal cut-off value of the LMR for evaluating the prognoses of GBC patients, a ROC curve analysis was performed. The optimal LMR cut-off value was found to be 2.685. We found that LMR, lymphocyte count, and monocyte count were potential surrogate prognostic markers. However, LMR had the best accuracy and robustness for predicting the mortality of GBC patients. When the correlations between LMR and clinicopathological characteristics were analyzed, we found that a low LMR was significantly associated with elderly age, a poorly differentiated tumor, palliative cholecystectomy, and TNM stage. The explanation for these correlations might be that elderly and terminal cancer patients are prone to have a poor immunological status and to suffer from inflammatory response due to the loss of physical function and appetite.^[30,31]^ Moreover, we found that LMR was significantly decreased in patients who had undergone a palliative l cholecystectomy.

Although the LMR is a useful biomarker for predicting the clinical outcomes of patients with a variety of malignancies, the underlying mechanism of its prognostic relevance remains uncertain. Lymphocyte, which is basic component of the innate immune system and the cellular basis of immunosurveillance and immunoediting,^[32]^ can destroy residual tumor cell and micro metastase and inhibit the proliferation and migration of tumor cell through activating an antitumor immune response.^[33]^ Additionally, lymphocytopenia is a common characteristic of patients with advanced cancers, including those in whom tumor cells have invaded the vasculature or lymph nodes or who have distant metastases.^[34]^ Lymphocytopenia has even proven to be an independent predictor of an adverse clinical outcome, which was corroborated by the results of our investigation.^[35,36]^ In contrast, inflammation can trigger the mobilization of monocyte to the peripheral blood and promote their differentiation into tumor-associated macrophages after they have been recruited to tumor tissues.^[37,38]^ Therefore, an increase in the circulating monocyte count may indicate the increased production of tumor-associated macrophages. Tumor-associated macrophages interact with cancer cells and promote tumor progression by producing various cytokines and chemokines, such as IL-6 and tumor necrosis factor-α.^[39,40]^ In addition, monocytes and their progeny have immunosuppressive, which also promotes tumor angiogenesis, tumor-cell invasion, and metastasis.^[41]^ Similar to the results of prior studies,^[42]^ a strong association between the pretreatment monocyte count and a poor prognosis for GBC patients was found in this study. The LMR is the ratio between the numbers of peripheral blood lymphocytes and monocytes. A high LMR indicates an increased lymphocyte count and/or a decreased monocyte count. An association has been found between the LMR and the prognosis of patients with various types of malignant neoplasms. In the course of our investigation, we found that the LMR was a superior independent predicator of the OS of patients with GBC compared with only the lymphocyte count or monocyte count. Additionally, the LMR is less susceptible to measurement variability due to conditions such as dehydration or fluid retention because it is a ratio instead of an absolute value.^[43]^

We have demonstrated that the LMR can be used as a prognostic marker for predicting the clinical outcomes of postsurgical GBC patients. However, the findings of the present study should be interpreted in consideration of its possible limitations. First, this was a retrospective study with a large sample of subjects, and some of the preoperative or follow-up data were incomplete, which may have had a negative impact on the survival analysis. Second, although the optimal cut-off value was determined based on ROC curve analysis in our study, there is no uniform optimal cut-off value for the LMR. Hence, the optimal cut-off value for GBC remain to be determined by multicenter prospective clinical studies. Additionally, because improvements in surgical and early-detection techniques, as well as in surgical efficacy and safety, are continually being made, the long-range data-accrual period of this study might have introduced bias. Therefore, a multicenter clinical study should be performed to confirm our findings.

## 5 Conclusion

Our study demonstrated that the pretreatment LMR was an independent prognostic factor for the clinical outcomes of postsurgical GBC patients. Furthermore, the predictive nomogram might be included in the routine assessment of GBC patients.

## Author contributions

Yan Deng, Zhen-Gang Sun, and Shuai Wang designed the study and performed the data analysis and drafted the manuscript. Ming-Fang Xu performed the data analysis, and drafted the manuscript. Feng Zhang, Xiao Yu, and Xue-Wen Zhang participated in the data analysis and drafted the manuscript. All authors participated in the data acquisition and manuscript revising. All authors approved the final manuscript to be submitted for publication.
